# Efficacy of Static Stretching Along With Frozen Tennis Ball Exercises in Individuals With Plantar Fasciitis

**DOI:** 10.7759/cureus.114000

**Published:** 2026-08-05

**Authors:** Vaibhav V Shinde, Pragati R Patil

**Affiliations:** 1 Department of Physiotherapy, Krishna College of Physiotherapy, Krishna Vishwa Vidyapeeth (Deemed to be University), Karad, IND

**Keywords:** cryotherapy, frozen tennis ball, plantar fasciitis, plantar heel pain, static stretching

## Abstract

Background: Plantar fasciitis, a prevalent kind of heel pain that affects people of all ages and involves inflammation of the fascia covering the plantar region, is a typical ailment in India. Physiotherapy is among the many available treatment choices. When combined, stretching and freezing tennis ball workouts will aid in recovery from the condition.

Methodology: The 30 participants were selected using a random sampling technique in accordance with the inclusion and exclusion criteria. A control group and an experimental group were established. A Foot Function Index (FFI) and a Numerical Pain Rating Scale (NPRS) were used for the pre-test evaluations. The patient received the regimen for two months. A post-test evaluation was then completed. The findings were determined using statistics and paired and unpaired testing.

Results: Thirty participants were divided equally into Group A (control, static stretching, *n* = 15) and Group B (experimental, frozen tennis ball + stretching, *n* = 15). Within-group analysis showed a significant reduction in NPRS scores in Group A (pre: 7.6 ± 1.88 vs. post: 6.73 ± 1.38; *t* = 4.516, *P* = 0.0005) and Group B (pre: 7.73 ± 1.75 vs. post: 3.53 ± 1.06; *t* = 16.039, *P* < 0.0001). FFI scores also improved significantly in Group A (pre: 66.0 ± 12.43 vs. post: 58.73 ± 11.82; *t* = 11.294, *P* < 0.0001) and Group B (pre: 70.73 ± 12.10 vs. post: 39.73 ± 5.69; *t* = 14.025, *P* < 0.0001). Between-group comparison of the post-test scores revealed a significant advantage for Group B over Group A in both NPRS (*t* = 7.099, *P* < 0.0001) and FFI (*t* = 5.609, *P* < 0.0001).

Conclusions: The findings indicate that static stretching and frozen tennis ball workouts have shown a significant and clinically beneficial effect on reducing plantar heel discomfort and impairment of foot function, particularly in individuals diagnosed with plantar fasciitis.

## Introduction

A major common and crippling musculoskeletal disorder affecting the lower limb is plantar fasciitis, which is characterized by pain and discomfort that is restricted to the proximal plantar fascia and inferior heel. The phrase *plantar fasciosis* has become more common in the clinical and research literature to more accurately reflect the underlying pathological changes because the condition is often described as a degenerative process rather than a purely inflammatory one, involving microtears, collagen disorganization, and fibroblastic proliferation within the plantar fascia.

Plantar fasciitis is a chronic and frequently recurrent condition, with a reported prevalence of 54% among affected individuals in an Indian cohort [1]. Given its chronicity and tendency for recurrence, a variety of conservative treatment approaches have been considered, with the primary objectives being pain relief, restoration of function, and correction of the underlying biomechanical deficiencies.

An essential biomechanical component that supports the foot's longitudinal arch and encourages proper gait mechanics is the plantar fascia. It is a thick covering of fibrous tissue that inserts at the base of each of the five toes' proximal phalanges after extending distally from the medial calcaneal tubercle [2]. Sharp, stabbing heel pain that is usually most intense in the initial moments following awakening in the morning or after extended times of relaxation is the hallmark clinical presentation of this condition. This phenomenon is explained by the contracture of the fascia during inactivity followed by abrupt stretching upon weight-bearing [3].

After prolonged standing, walking, or physical exercise, this soreness may recur, although it usually goes away with persistent ambulation [4]. Both mechanical overloading and systemic metabolic factors may contribute to the pathogenesis of the condition, which disproportionately affects people in their fourth and sixth decades of life. It is commonly seen in athletic populations, especially long-distance runners, as well as in sedentary people and those who are overweight [5].

A fascia thickness greater than 4 mm is a reliable ultrasonographic marker of plantar fasciitis, supporting clinical diagnosis and directing treatment planning. As a result, diagnostic imaging, especially musculoskeletal ultrasonography, has been utilized more often to objectively assess plantar fascia thickness and morphology [6].

The therapeutic use of cryotherapy has gained interest as an additional treatment option for plantar fasciitis, in addition to stretching. Cryotherapy reduces local inflammation, inhibits nociceptive nerve transmission, and modifies the tissue healing response, among other processes. Static stretching combined with cryo-massage utilizing a frozen tennis ball is a practical and approachable combination; it could be utilized in both home-based and clinical settings [7].

Using a frozen tennis ball is a useful and affordable way to apply cryotherapy to the plantar surface. As the foot rolls over the ball, it simultaneously offers the benefits of cryotherapy and a type of plantar fascia massage. When combined with static stretching, this dual-action intervention may have a synergistic therapeutic effect by mechanically mobilizing the plantar fascia, stimulating blood flow after the cold application, and providing myofascial release to the intrinsic musculature of the foot [8]. Stretching exercises that target the gastrocnemius-soleus complex and the plantar fascia are among the most well-researched and clinically recommended therapies [9].

Given the growing body of evidence demonstrating the effectiveness of static stretching in improving tissue extensibility, reducing fascial tension, and increasing ankle joint range of motion, all of which address the biomechanical abnormalities associated with the condition, it has become a key component of conservative management. Both calf stretching and plantar fascia-specific stretching protocols result in clinically significant pain reductions and improvements in function in people with plantar fasciitis, according to a systematic review and meta-analysis, supporting their regular inclusion in rehabilitation programs.

There is substantial therapeutic value to a functional and fundamental structural continuity between the Achilles tendon and plantar fascia. Research has demonstrated that extending the Achilles tendon and plantar fascia at the same time produces better outcomes than stretching either structure independently because of the tensional interdependence of the posterior kinetic chain [10].

Similar to this, a gastrocnemius-soleus stretching program has been proven to be a successful therapeutic intervention for plantar fasciitis; structured stretching regimens that target these muscle groups significantly reduce pain intensity and improve foot function. The combined effects of static stretching and frozen tennis ball exercises as a combination therapy strategy for plantar fasciitis, despite the increasing amount of evidence demonstrating the effectiveness of individual conservative therapies.

The heterogeneity of previous research and the requirement for trials that investigate combination therapy in standardized protocols were brought to light by a thorough systematic review of physiotherapeutic approaches for plantar fasciitis. Thus, the goal of the existing research is to assess how well static stretching and frozen tennis ball workouts affect plantar fascia thickness, discomfort, and function in people with plantar fasciitis.

## Materials and methods

Study design

The present study was an experimental research study conducted to compare the effects of the two interventions among the selected participants. The study was carried out in Satara District over the period from October 2023 to April 2024 (i.e., six months). Ethical clearance for the study was obtained from the Ethics Committee and Protocol Committee before the commencement of data collection. A total of 30 participants who fulfilled the inclusion criteria were enrolled and divided into two equal groups, with 15 participants in each group, using a simple random sampling method.

Selection of subjects

The sample size for the study was calculated using the formula:

$n=\frac{Z^{2}pq}{L^{2}}$

and participants were recruited from Satara District [11]. Subjects were enrolled in the study based on the predetermined inclusion and exclusion criteria. Before any study-related procedures, each participant was given a detailed explanation of the aims and objectives of the study, following which written informed consent was obtained. Demographic and contact details of each participant, including name, age, gender, and email address, were recorded on a case sheet.

Inclusion criteria

Male and female participants aged 20 to 50 years were included in the study if they had a positive Windlass test and a clinical diagnosis of plantar fasciitis, as well as if they reported painful first steps in the morning, a typical clinical symptom of the condition [12].

Exclusion criteria

Exclusion criteria for the study included participants whose plantar fasciitis was attributable to an underlying systemic disease, as this could confound the treatment outcomes. Participants with any associated foot pathology, those who had previously undergone ankle or foot surgery, or those with a history of foot or ankle injury were also excluded. Additionally, participants were not eligible for the trial if they had a congenital foot deformity.

Procedure

The research was carried out on the basis of a pre-designed research protocol. The 30 participants were selected and randomly divided into two groups: Group A (control group, n = 15) and Group B (experimental group, n = 15). The intervention program consisted of 32 sessions for both groups, administered 4 times per week over a period of two months, with each session lasting 45 to 50 minutes. A pre-test assessment of pain, plantar fascia integrity, and foot function was carried out just before the commencement of the protocol using the Numerical Pain Rating Scale (NPRS) [13], the Windlass test [14], and the Foot Function Index (FFI) [15]. After the two-month intervention program, post-test assessments were repeated using the same outcome measures to evaluate the pre- and post-treatment effectiveness of the protocol.

Treatment protocol

The treatment protocol was specifically designed and progressively structured for each group. Group A received conventional static stretching, while Group B received an experimental protocol comprising frozen tennis ball cryotherapy combined with a progressive stretching program. Exercises in Group B progressed weekly in terms of repetitions, sets, and hold times throughout the two-month intervention. The detailed treatment protocol for both groups is presented in Table 1.

**Table 1:** Treatment protocol: Group A versus Group B.

Parameter	Group A - Control group (*n* = 15)	Group B - Experimental group (*n* = 15)
Intervention	Conventional static stretching	Frozen tennis ball exercises + stretching progression
Sessions	32 sessions (4 sessions/week × 2 months)	32 sessions (4 sessions/week × 2 months)
Duration	45-50 minutes per session	45-50 minutes per session
Electrotherapy	Ultrasound: 1 MHz for 8 minutes	Ultrasound: 1 MHz for 8 minutes
Exercise 1	Plantar fascia stretches - 30 seconds hold × 3 sets	Frozen ice ball rolling - 15 minutes
Exercise 2	Gastrocnemius stretch - 30 seconds hold × 3 sets	Gastrocnemius and soleus stretch - 30 seconds hold × 3 sets
Exercise 3	Soleus stretch - 30 seconds hold × 3 sets	Passive toe stretch - 30 seconds hold × 3 sets
Exercise 4	Passive hamstring stretches - 30 seconds hold × 3 sets	Passive hamstring stretches -30 seconds hold × 3 sets
Exercise 5	Stair stretch	Stair stretches and marble pickup
Progression	No weekly progression described	Weekly progression in repetitions (sets) and hold times throughout the two-month program

Selection of outcome measures

The outcome measures listed below were chosen to evaluate pain intensity and foot function before and after the intervention.

Numerical Pain Rating Scale

The NPRS was utilized to assess pain intensity. It is an 11-point numerical scale ranging from 0 to 10, where 0 represents "no pain," and 10 represents "worst possible pain." Participants were instructed to choose the number that most accurately reflected how much pain they were experiencing at the time [13].

Foot Function Index

The FFI was administered to assess the impact of foot pathology on function in terms of pain, disability, and activity restriction. It is a self-administered index comprising 23 items across three subscales, generating both subscale and total scores [15].

Statistical analysis

Data analysis was performed using SPSS version 26.0 (IBM Corp., Armonk, NY). Within-group comparisons were performed using a paired t-test, while between-group comparisons were performed using an unpaired t-test. Data normality was assessed using the Shapiro-Wilk test before applying statistics.

## Results

Thirty people with a properly assessed diagnosis of plantar fasciitis participated in the current study and were randomly divided into two groups: Group B (experimental group, *n* = 15) received frozen tennis ball activities in addition to progressive stretching, while Group A (control group, *n* = 15) received traditional static stretching. The outcome measures were evaluated to conduct assessments both before and after the intervention.

After eight weeks of intervention, the pre-test score for Group A was 7.6 ± 1.882, and it decreased to 6.733 ± 1.38. In Group B, the pre-test score was 7.733 ± 1.751; however, the post-test evaluation showed a decline to 3.533 ± 1.060. Notably, both groups experienced a significant difference in pain intensity after treatment, but Group B (*t* = 16.03, *P* < 0.0001) showed a more noticeable decrease than Group A (*t* = 4.514, *P* < 0.0005). The average pain scores for Groups A and B before and after the intervention can be seen in Table 2.

**Table 2:** Comparison of mean NPRS scores within and between the two groups. Values are expressed as mean ± SD. Statistical analysis was performed using the paired t-test. Statistical significance was considered at *P* < 0.05. NPRS, Numerical Pain Rating Scale

NPRS	Pre	Post	*t*-value	*P*-value
Group A	7.6 ± 1.882	6.7333 ± 1.38	4.516	<0.0001
Group B	7.7333 ± 1.751	3.5333 ± 1.060	16.039	<0.0001

Group A's pre-test score reached 66 ± 12.43, and it lowered to 58.733 ± 11.823 after a period of eight weeks of intervention. The pre-test score for Group B was 70.733 ± 12.104; however, the post-test evaluation revealed a reduction to 39.733 ± 5.688. After therapy, there was a significant variation in the FFI between the two groups; nevertheless, Group B's FFI decreased more noticeably (*t* = 14.025, *P* < 0.0001) than Group A's (*t* = 11.294, *P* < 0.0001). The mean FFI for Groups A and B both before and following the intervention can be seen in Table 3.

**Table 3:** Comparison of mean FFI scores within and between the two groups. Values are expressed as mean ± SD. Statistical analysis was performed using the paired t-test. Statistical significance was considered at *P* < 0.05. FFI, Foot Function Index

FFI	Pre	Post	*t*-value	*P*-value
Group A	66 ± 12.43	58.733 ± 11.823	11.294	<0.0001
Group B	70.7333 ± 12.104	39.733 ± 5.688	14.025	<0.0001

After an eight-week intervention, Group A's post-test score was 6.733 ± 1.387, while Group B's post-test score was 3.533 ± 1.060, with a *t* score of 7.099 and *P* < 0.0001. However, the post-test evaluation stated that Group B, the experimental group, experienced a greater decline in pain compared with Group A, the control group. The average pain ratings for Groups A and B following the intervention are described in Table 4.

**Table 4:** Comparison of post-test scores of NPRS of Group A and Group B. Values are expressed as mean ± SD. Statistical analysis was performed using the unpaired t-test. Statistical significance was considered at *P* < 0.05. NPRS, Numerical Pain Rating Scale

NPRS	Post	*t*-value	*P* -value
Group A	6.73 ± 1.387	7.099	<0.0001
Group B	3.53 ± 1.060		

Group A's final test result after eight weeks of intervention was 58.733 ± 11.823, and Group B's post-test score was 39.733 ± 5.688, with a *t* score of 5.609 and *P* < 0.0001. Even so, the post-test evaluation presented that Group B, the experimental group, exhibited a greater improvement in foot function than Group A, the control group. The mean FFI scores for Groups A and B following the intervention are as seen in Table 5.

**Table 5:** Comparison of post-test FFI scores between Group A and Group B. Values are expressed as mean ± SD. Statistical analysis was performed using the unpaired t-test. Statistical significance was considered at *P* < 0.05. FFI, Foot Function Index

FFI	Post	*t*-value	*P*-value
Group A	58.73 ± 11.823	5.609	<0.0001
Group B	39.73 ± 5.688		

## Discussion

The effectiveness of static stretching in conjunction with frozen tennis ball workouts for those with plantar fasciitis was examined in this study. Following the intervention, both the NPRS and the FFI ratings showed statistically noteworthy gains in Group B, which underwent both frozen tennis ball activities and static stretching. The literature currently available on multimodal treatment techniques for the treatment of plantar fasciitis supports and is consistent with these findings.

The frozen tennis ball workout directly targets the intrinsic foot musculature and plantar fascia, acting as a combination of cryotherapy and self-myofascial release. Group B of the current study showed a significant improvement in NPRS scores, which is consistent with the pain-reducing mechanisms outlined by Ajimsha et al. [8]. These mechanisms effectively reduce nociceptive sensitization and promote tissue extensibility when mechanical stimulation is combined with local cooling.

In the current study, two groups were given a static stretching routine that included gastrocnemius-soleus and plantar fascia-specific stretching activities. Siriphorn and Eksakulkla's findings are further supported by the additional benefit seen in Group B over stretching alone, demonstrating that clinically superior results in FFI scores are obtained when stretching is combined with mechanical and thermal stimulation through the frozen tennis ball [9].

Multimodal therapy that combines stretching with supplementary physical modalities generally yields greater results than unimodal approaches, according to a comprehensive assessment of physiotherapeutic treatments for plantar fasciitis. This finding is strongly supported by the notable improvement in both NPRS and FFI in Group B of the current investigation. The frozen tennis ball exercise, by providing simultaneous cryotherapy and mechanical pressure in relation to the plantar fascia, addresses multiple pathophysiological contributors to plantar fasciitis, such as local inflammation, restricted fascial mobility, and reduced intrinsic muscle flexibility. This is consistent with Boob et al.'s multimodal treatment approach [12].

Similar principles are embodied in the frozen tennis ball training routine used in Group B of the current study: the rolling movement dynamically stretches and mobilizes the plantar fascia, while the cold surface of the frozen ball provides cryotherapeutic input. According to Jadhav and Gurudut, the improved results observed in Group B's NPRS and FFI scores are strongly supported both theoretically and empirically by the similarities between the mechanisms of cryostretch and the frozen tennis ball intervention [16].

Ranbhor et al. evaluated the immediate effects of foam roller treatment on ankle range of motion and pain in patients with plantar fasciitis in a randomized controlled experiment. They discovered that foam rolling greatly reduced ankle discomfort and dorsiflexion. The basic mechanism of mechanical decompression and plantar fascia stimulation is similar to rolling a frozen tennis ball, even though the myofascial release tool utilized in this study was a foam roller [17].

Collectively, the findings of the present study, demonstrating significant improvements in NPRS and FFI in Group B receiving frozen tennis ball exercises alongside static stretching, are well-corroborated by current evidence. The frozen tennis ball acts as a cost-effective, easily accessible self-treatment tool that integrates the benefits of cryotherapy, myofascial release, and dynamic stretching. These combined effects address both the pain and functional limitations characteristic of plantar fasciitis more comprehensively than stretching alone, supporting its incorporation as an adjunct modality in the physiotherapeutic management of this condition.

Limitations

The study's small sample size and brief intervention period may have limited the findings' applicability to a larger group of people with plantar fasciitis. It was difficult to determine if the improvements in NPRS and FFI scores continued after the course of treatment due to the absence of long-term follow-up assessments. Additionally, the study did not take into consideration the patients' body mass index or level of physical activity, both of which are known to affect how severe plantar fasciitis symptoms are.

Strengths

The NPRS and FFI, two proven and trustworthy instruments for evaluating pain and functional impairment in plantar fasciitis, were used in the study's randomized controlled design. A low-cost, easily repeatable, and patient-friendly modality, the frozen tennis ball exercise intervention improves the findings' clinical applicability and translational usefulness. A controlled basis for assessing the additional therapeutic benefit of the frozen tennis ball exercise was provided by the inclusion of a comparison group that received stretching alone.

Future recommendations

To properly assess the sustainability of treatment benefits, longer follow-up studies should be a part of future studies with periods of three to six months or longer, and bigger, more varied sample groups. To determine the frozen tennis ball exercise's relative effectiveness within the range of self-care therapies, comparative studies comparing it to other myofascial release tools like foam rollers or massage balls would be beneficial. Future research should also consider objective biomechanical outcome measures, such as plantar pressure distribution and ultrasound-measured fascial thickness, to provide a more comprehensive assessment of tissue-level alterations following intervention.

## Conclusions

The study's findings indicate that both the intervention group and the control group demonstrated statistically significant improvements in foot function and pain intensity following an eight-week intervention. However, the intervention group, which combined gradual static stretching with frozen tennis ball movements, showed noticeably better results. By concurrently addressing local inflammation, nociceptive sensitization, and fascial restriction, the dual therapeutic action of the frozen tennis ball, which combines cryotherapy with myofascial release and plantar fascial mobilization, seems to synergistically enhance the advantages of static stretching. It is concluded that the inclusion of frozen tennis ball exercise in the conservative physiotherapeutic treatment of plantar fasciitis is a clinically successful, economical, and readily self-administered adjunct modality.
